# Ovarian Reserve Markers in Premature Ovarian Insufficiency: Within Different Clinical Stages and Different Etiologies

**DOI:** 10.3389/fendo.2021.601752

**Published:** 2021-03-18

**Authors:** Xue Jiao, Tingting Meng, Yiwei Zhai, Lijuan Zhao, Wei Luo, Peihao Liu, Yingying Qin

**Affiliations:** ^1^ Center for Reproductive Medicine, Cheeloo College of Medicine, Shandong University, Jinan, China; ^2^ National Research Center for Assisted Reproductive Technology and Reproductive Genetics, Shandong University, Jinan, China; ^3^ Key Laboratory of Reproductive Endocrinology of Ministry of Education, Shandong University, Jinan, China; ^4^ Shandong Provincial Clinical Medicine Research Center for Reproductive Health, Shandong University, Jinan, China

**Keywords:** premature ovarian insufficiency (POI), premature ovarian failure (POF), ovarian reserve, clinical staging, etiology

## Abstract

**Objective:**

To characterize the ovarian reserve indicators for premature ovarian insufficiency (POI) at different disease stages and with various etiologies.

**Methods:**

According to different FSH levels and menstrual conditions, patients with normal ovarian reserve (NOR with 5 IU/L<FSH<10 IU/L, n=987), precursor stage of POI (pre-POI with 10 IU/L<FSH ≤ 25 IU/L, n=410), early POI (25 IU/L<FSH ≤ 40 IU/L n=147), and premature ovarian failure (POF with FSH>40 IU/L, n=454) were retrospectively screened and their records were abstracted from Reproductive Hospital Affiliated to Shandong University between 2014 and 2019. Based on the known etiologies, POI patients were subdivided into genetic, iatrogenic, autoimmune and idiopathic subsets according to the known etiologies. The phenotypic features were compared within different subgroups, and the predictive value of ovarian reserve markers was analyzed.

**Results:**

The ovarian reserve indicators consecutively deteriorated with the progress of ovarian insufficiency, indicated as an increase of FSH and LH but decrease of AMH, inhibin B, AFC, E_2_ and T (P<0.01). Most of them changed significantly from NOR to pre-POI while remained relatively stable at a low level or even undetectable at early POI and POF stage. AMH showed the highest predictive value for pre-POI (AUC 0.932, 95% CI 0.918-0.945) and POI (AUC 0.944, 95% CI 0.933-0.954), and the combination of AMH and AFC was highly promising for early prediction. Additionally, significant differences existed in AMH, inhibin B and AFC among women with different etiologies of POI (P<0.05), and the genetic POI presented the worst hormone status.

**Conclusions:**

Our study indicated a high heterogeneity of POI in both endocrine hormones and etiological phenotypes. The quantitative changes and cutoff values of AMH and AFC could provide new insights in the prediction and early diagnosis of POI.

## Introduction

Premature ovarian insufficiency (POI) is a common reproductive endocrine disorder defined by the cessation of ovarian function before the age of 40. POI is clinically characterized by oligomenorrhea or amenorrhea with increased gonadotrophins (FSH>25 IU/L) and decreased estradiol (E2) (1). Ovarian insufficiency is a continuum of impaired ovarian function or ovarian aging rather than a specific dichotomous state. This condition can be transient or progressive, and usually results in eventual premature menopause (2, 3). According to different FSH levels, fecundity, and menstrual status, POI has been subdivided into three consecutive but progressive stages: occult, biochemical, and overt ovarian insufficiency (3). Premature ovarian failure (POF) is thus considered as the end stage of POI with FSH>40 IU/L. However, the evidence-based progression of POI is still lacking.

Either a small primordial follicle pool or rapid follicle exhaustion is associated with ovarian dysfunction and fecundity decline and results in POI (2, 4). Currently, the main ovarian reserve indicators widely used in clinics include FSH, E_2_, inhibin B, Anti-Müllerian hormone (AMH), and antral follicle count (AFC) (5). Among these factors, FSH is the single one used for POI diagnosis but limited by its high inter- or intra-cycle variability (6). AMH and AFC have recently been considered more promising for assessing ovarian reserve, given their high sensitivity and specificity in predicting ovarian response and good inter-cycle reliability (7–9). Secreted mainly by small antral follicles, inhibin B is the most commonly utilized marker for ovarian activity rather than ovarian reserve (10, 11). However, none of these markers has been proven to be optimal for predicting the residual follicle pool and reproductive lifespan. Their dynamics and correlations during the progressive ovarian insufficiency still remain unclear.

POI is highly heterogeneous in etiology. A wide spectrum of causes has been considered, including genetic, autoimmune, or iatrogenic. Irrespectively, the majority remains to be elucidated (12). POI patients with different etiologies presented distinct phenotypes and endocrine hormones. Patients with genetic etiologies had the most severe disease phenotype compared to those with other etiologies (13). Whereas, Falorni et al. found that patients with autoimmune POI showed significantly higher inhibin B and AMH than those with idiopathic POI (14, 15). Therefore, whether ovarian reserve markers could be potential indicators for etiology classification in women with POI needs further exploration.

In the current study, ovarian reserve indicators were characterized in POI patients with different etiologies at different stages, and their significance in predicting POI progress and classifying different etiologies were further evaluated.

## Materials and Methods

### Patients

A total of 1998 women less than 40 years old with different ovarian reserve were retrospectively screened, and their records were abstracted from the Reproductive Hospital Affiliated to Shandong University between July 2014 and July 2019. The study was approved by the Ethics Committee of Reproductive Medicine of Shandong University. Written informed consents were obtained from all participants.

According to the FSH level and menstrual conditions, all participants were sub-grouped into normal ovarian reserve (NOR, n=987), pre-POI (n=410), early POI (n=147), and POF (n=454) group. Women with regular menstrual cycles and normal endocrine hormones (5 IU/L<FSH<10 IU/L and 1.2 ng/mL<AMH<4.7 ng/mL), who sought infertility treatment due to tubal or male factors, were included as NOR. Women with regular or irregular menses and high FSH level (10 IU/L<FSH ≤ 25 IU/L, on two occasions >4 weeks apart) was considered as the precursor stage of POI and defined as pre-POI in our study. The diagnostic criteria of POI included oligo/amenorrhea for at least 4 months, and elevated FSH level >25 IU/L (on two occasions >4 weeks apart), among which women with 25 IU/L<FSH ≤ 40 IU/L were defined as early POI and FSH>40 IU/L as POF. Women with polycystic ovarian syndrome and hyperprolactinemia were excluded. According to known etiologies, patients with POI were subdivided into four groups: genetic POI, iatrogenic POI, autoimmune POI and idiopathic POI, as previously reported (13). Patients with chromosomal abnormalities (61/601, 10.15%) were included into genetic group, including 23 X-structural abnormalities, 22 X-numerical abnormalities, 9 X-autosomal translocations, 3 autosomal abnormalities and 4 (45,X/46,XY) mosaicism. Patients with ovarian surgery (31 ovarian cystectomy, 2 oophorectomy) and chemo-radiotherapy (2 cases) were included into iatrogenic group. Patients with autoimmune disease, including hypothyroidism or Hashimoto thyroiditis (n=25), psoriasis (n=1), rheumatoid arthritis (n=2), multiple sclerosis (n=1), dermatomyositis (n=1) or positive for thyroid antibodies (n=72) as immune POI. Ultimately, patients with unknown causes were classified as idiopathic POI (n=403).

### Hormone Measurement and Ultrasonography

Peripheral blood was sampled on day 2-4 of the menstrual cycle or randomly (for women not menstruating frequently). Endocrine hormones FSH, LH, prolactin (PRL), E_2_, and testosterone (T) were detected through chemiluminescence immunoassay (Roche Diagnostics, Mannheim, Germany). AMH and inhibin B were detected by enzyme-linked immunosorbent assay (Kangrun Biotech, Guangzhou, China). The intra- and inter-assay coefficients of variation were <10% and <15%, respectively. Transvaginal ultrasonography was routinely conducted. AFC was defined as the number of bilateral follicles (2-10 mm in diameter) in early follicular phase.

### Karyotype Analysis

Karyotype analysis was performed on GTG-banded metaphase chromosomes prepared from peripheral lymphocyte cultures, using a standard protocol that generated 400-450 band resolutions. Chromosome polymorphisms were recorded but classified as normal (16).

### Statistical Analysis

SPSS 23.0 (SPSS Inc., Chicago, IL) was used for statistical analysis. The single-sample Kolmogorov-Smirnov test was used for normality of distribution. Continuous data in normality distribution were expressed as mean ± standard deviation and compared by Student t-test or one-way analysis of variance. Continuous variables that were not normally distributed were presented as median (quartile interval) and compared by nonparametric test. Kruskal-Wallis ANOVA and multiple logistic regression were used for multiple comparisons, and P value was corrected by Bonferroni adjustment. The independent and combined predictive analyses were performed by binary logistic regression analysis and receiver-operator characteristic (ROC) curve. P<0.05 was considered statistically significant.

## Results

### Baseline and Reproductive Characteristics

The records of 1998 women aged before 40 years old were retrospectively abstracted, including 987 women with NOR, 410 pre-POI, 147 early POI and 454 POF. The age at diagnosis significantly varied among pre-POI, early POI and POF groups (32.38 ± 4.07 y vs. 30.22 ± 3.72 y vs. 29.55 ± 4.19 y, P<0.001), indicating both women with early POI and POF presented with much earlier onset of ovarian insufficiency (P<0.001). Age at menarche was comparable among women with NOR, pre-POI and early POI (14.07 ± 1.46 y vs. 14.08 ± 1.84 y vs. 14.25 ± 2.05 y, all P>0.999), except that women with POF (14.92 ± 2.57 y) experienced significantly later menarche (all P<0.05). In addition, there were 131 cases (31.95%) with irregular menses in pre-POI group. Women with early POI and POF showed an earlier age of irregular menstruation (~ 2 years) compared with women in pre-POI (21.52 ± 5.71 y vs. 21.70 ± 5.89 y vs. 23.65 ± 7.38 y, P=0.007). There was no difference of age at amenorrhea among women with early POI and POF (22.59 ± 5.67 y vs. 23.24 ± 5.82 y, P=0.417). Women among four groups also showed different body mass index (BMI) (P=0.040), with difference mainly existing between patients with early POI and POF (23.30 ± 3.50 y vs. 22.44 ± 3.20 y, P=0.044). The baseline and reproductive characteristics of the participants were summarized in **Table 1** and **Supplemental Table 1**.

**Table 1:** Characteristics of 1998 women.

Characteristics	NOR	pre-POI	Early POI	POF	*P*	*P-adj^i^*
N	987	410	147	454	–	–
Age at diagnosis (y)^d^ ^,^ ^e^	–	32.38 ± 4.07	30.22 ± 3.72	29.55 ± 4.19	<0.001^g^	–
BMI (kg/m^2^)^f^	22.81 ± 3.24	22.84 ± 3.43	23.30 ± 3.50	22.44 ± 3.20	0.040^g^	–
Age at menarche (y)^c^ ^,^ ^e^,^f^	14.07 ± 1.46	14.08 ± 1.84	14.25 ± 2.05	14.92 ± 2.57	<0.001^g^	–
Age at irregularity (y)^e^	–	23.65 ± 7.38	21.52 ± 5.71	21.70 ± 5.89	0.007^g^	–
Age at amenorrhea (y)	–	–	22.59 ± 5.67	23.24 ± 5.82	0.417^g^	–
FSH (IU/L)^a^ ^,^ ^b^ ^,^ ^c^ ^,^ ^d^ ^,^ ^e^ ^,^ ^f^	6.81 ± 1.17	14.90 ± 4.23	31.73 ± 4.35	70.77 ± 23.11	<0.001^g^	<0.001
LH (IU/L)^a^ ^,^ ^b^ ^,^ ^c^ ^,^ ^d^ ^,^ ^e^ ^,^ ^f^	4.63 ± 1.70	6.53 ± 3.23	16.37 ± 8.28	35.65 ± 13.90	<0.001^g^	<0.001
FSH/LH Ratio^a^ ^,^ ^b^ ^,^ ^c^ ^,^ ^e^ ^,^ ^f^	1.64 ± 0.66	2.49 ± 1.22	2.37 ± 1.14	2.14 ± 0.84	<0.001^g^	<0.001
PRL (ng/mL)	14.32 (10.15, 20.00)	13.59 (9.90, 19.50)	11.41 (7.75, 15.90)	9.95 (7.39, 14.52)	<0.001^h^	0.268
E_2_ (pg/mL)^b^ ^,^ ^c^ ^,^ ^d^ ^,^ ^e^ ^,^ ^f^	34.30 (25.30, 44.90)	30.20 (19.50, 45.28)	15.65 (5.23, 31.60)	7.15 (5.00, 20.54)	<0.001^h^	<0.001
T (ng/dL)^b^ ^,^ ^c^ ^,^ ^e^	21.18 ± 9.67	20.55 ± 11.38	18.39 ± 12.16	18.14 ± 13.00	<0.001^g^	0.002
AMH (ng/mL)^a^ ^,^ ^b^ ^,^ ^c^ ^,^ ^d^ ^,^ ^e^	2.513 (1.817, 3.491)	0.424 (0.091, 1.024)	0.078 (0.071, 0.091)	0.078 (0.071, 0.087)	<0.001^h^	<0.001
Inhibin B (pg/mL)^a^ ^,^ ^b^ ^,^ ^c^ ^,^ ^d^ ^,^ ^e^	65.18 (45.04, 87.89)	37.05 (14.30, 65.07)	13.00 (10.00, 15.50)	13.30 (10.00, 16.00)	<0.001^h^	<0.001
AFC ^a^ ^,^ ^b^ ^,^ ^c^ ^,^ ^d^ ^,^ ^e^	8 (6, 14)	4 (1, 6)	0 (0, 2)	0 (0, 1)	<0.001^h^	<0.001

Data are expressed as the mean ± standard deviation or median (interquartile range).

BMI, body mass index; FSH, follicle-stimulating hormone; LH, luteinizing hormone; E_2_, estradiol; PRL, prolactin; T, testosterone; AMH, anti-müllerian hormone; AFC, antral follicle count.

a P<0.05 for the comparison between NOR and pre-POI.

b P<0.05 for the comparison between NOR and early POI.

c P<0.05 for the comparison between NOR and POF.

d P<0.05 for the comparison between pre-POI and early POI.

e P<0.05 for the comparison between pre-POI and POF.

f P<0.05 for the comparison between early POI and POF.

g One-way analysis of variance.

h Kruskal-Wallis ANOVA test.

i Adjusted P‐value after correcting for age and BMI through multiple logistic regression.

### Variation of Ovarian Reserve Markers at Different Stages of Ovarian Insufficiency

**Figure 1:**
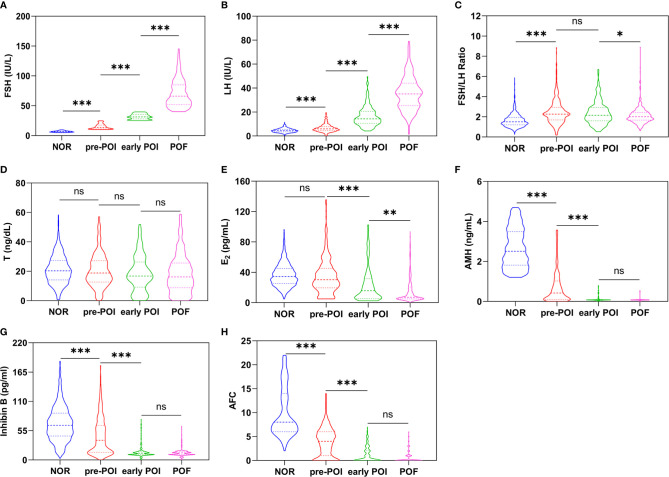
**(A–H)** show the variations of FSH, LH, FSH/LH Ratio, T, E2, AMH, inhibin B and AFC in different stages of ovarian insufficiently respectively. *P < 0.05; **P < 0.01; ***P < 0.001; ns, not significant.

As expected, the markers of ovarian reserve consecutively deteriorated as the progress of ovarian insufficiency, indicated as an increase of FSH and LH but a decrease of E_2_, T, AMH, inhibin B and AFC, even after correcting for age and BMI through multiple logistic regression (P<0.01) (**Table 1** and **Figure 1**). No difference of PRL was found among different groups (P=0.268). Intriguingly, FSH and LH showed different change patterns. FSH showed a 2-fold increase steadily in each stage along with ovarian function decline (6.81 ± 1.17 IU/L vs. 14.90 ± 4.23 IU/L vs. 31.73 ± 4.35 IU/L vs. 70.77 ± 23.11 IU/L, P<0.001), whereas LH remained normal at pre-POI stage but presented a 2.5-fold increase later in early POI and POF (4.63 ± 1.70 IU/L vs. 6.53 ± 3.23 IU/L vs. 16.37 ± 8.28 IU/L vs. 35.65 ± 13.90 IU/L, P<0.001). The FSH/LH ratio thus increased significantly from NOR to pre-POI (1.64 ± 0.66 vs. 2.49 ± 1.22, P<0.001), and later maintained high constantly. Similar to LH, no significant difference of E_2_ existed between patients with NOR and pre-POI (34.30 [25.30, 44.90] pg/mL vs. 30.20 [19.50, 45.28] pg/mL, P>0.999), and it showed a 2-fold decrease thereafter (15.65 [5.23, 31.60] pg/mL vs. 7.15 [5.00, 20.54], P<0.01). The level of T declined slowly along with POI progress (21.18 ± 9.67 ng/dL vs. 20.55 ± 11.38 ng/dL vs. 18.39 ± 12.16 ng/dL vs. 18.14 ± 13.00 ng/dL, P=0.002), and no significant difference existed between any two adjacent stages (all P>0.05).

The decrease pattern of AMH, inhibin B and AFC was quite similar, with significance among NOR, pre-POI and early POI stages (P<0.001) but comparable between early POI and POF (P>0.05). From NOR to pre-POI, AMH showed the most significant decline and relatively high sensitivity with approximately 6 times of decrease (from 2.513 ng/mL to 0.424 ng/mL), compared to an approximately 2-fold decline of inhibin B (from 65.18 pg/mL to 37.05 pg/mL) and AFC (from 8 to 4). Similarly, the three indicators decreased at least twice from pre-POI to POI. While in 601 patients with early POI and POF, AMH was undetectable in 75.04% (451/601), inhibin B in 70.38% (423/601) and AFC was invisible in 65.72% (395/601) of cases.

### The Predictive Value of Ovarian Reserve Markers on pre-POI and POI

**Figure 2:**
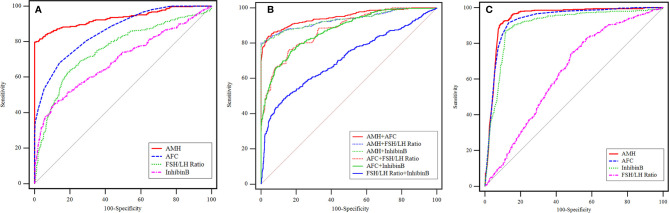
The ROC curves of AMH, AFC, inhibin B and FSH/LH ratio for pre-POI and POI prediction. **(A)** Single variable for pre-POI prediction. **(B)** Multivariable models for pre-POI prediction. **(C)** Single variable for POI prediction.

To explore the predictive value of ovarian reserve indicators for pre-POI and POI, AMH, inhibin B, AFC and FSH/LH ratio were further analyzed given their significant difference from NOR to pre-POI. The specificity and sensitivity of these markers were analyzed by ROC curves (**Tables 2**, **3** and **Figure 2**). In terms of predicting pre-POI, the cutoff values of AMH, inhibin B, AFC and FSH/LH ratio were 1.211 ng/mL, 31.74 pg/mL, 5 and 2.11, respectively. AMH showed the best predictive value (AUC 0.932, 95% CI 0.918-0.945) both in sensitivity and in specificity, followed by AFC (AUC 0.868, 95% CI 0.848-0.885) and FSH/LH ratio (AUC 0.749, 95% CI 0.726-0.772), whereas inhibin B with the most unsatisfactory accuracy (AUC 0.704, 95% CI 0.679-0.727) (**Figure 2A**). To determine whether a combination of markers is more promising for pre-POI prediction, we included these four markers for multivariable prediction models. Among the dual-indicator models, AMH plus AFC showed the highest predictive accuracy, with 84.08% in sensitivity and 95.68% in specificity (95% CI 0.935-0.959, P<0.001). When making pairwise comparison for all prediction models, we found that the combination of inhibin B or FSH/LH ratio with AMH single model or AMH + AFC dual model made no significant difference for predictive accuracy (P>0.05) (**Table 2**, **Supplemental Table 2** and **Figure 2B**).

**Table 2:** The ROC curve analysis for pre-POI prediction.

Variable	AUC	95% CI^b^	cutoff	Sensitivity	Specificity	P value
AMH	0.932	0.918-0.945	1.211 (ng/mL)	79.74%	99.80%	<0.001
AFC	0.868	0.848-0.885	5	69.53%	85.91%	<0.001
FSH/LH ratio	0.749	0.726-0.772	2.11	62.44%	81.05%	<0.001
Inhibin B	0.704	0.679-0.727	31.74 (pg/mL)	46.59%	89.06%	<0.001
AMH+AFC	0.948	0.935-0.959	–	84.08%	95.68%	<0.001
AMH + FSH/LH ratio	0.932	0.918-0.945	–	79.95%	99.90%	<0.001
AMH + Inhibin B	0.932	0.917-0.945	–	81.58%	97.67%	<0.001
AFC + FSH/LH ratio	0.884	0.866-0.900	–	77.83%	83.85%	<0.001
AFC + Inhibin B	0.880	0.862-0.897	–	77.40%	82.41%	<0.001
FSH/LH + Inhibin B	0.715	0.691-0.739	–	49.88%	86.83%	<0.001
AMH + AFC + FSH/LH ratio	0.948	0.935-0.959	–	84.04%	95.58%	<0.001
AMH + AFC + Inhibin B	0.948	0.935-0.959	–	84.88%	95.16%	<0.001

AMH, anti-müllerian hormone; AFC, antral follicle count; ROC, receiver-operator characteristic curve; AUC, the area under the curve. ^b^Binomial exact test.

**Table 3:** The ROC curve analysis for POI prediction.

Variable	AUC	95% CI^b^	cutoff	Sensitivity	Specificity	P value
AMH	0.944	0.933-0.954	0.250 (ng/mL)	92.46%	90%	<0.001
AFC	0.927	0.915-0.938	3	92.51%	85.21%	<0.001
Inhibin B	0.902	0.889-0.915	19.08 (pg/mL)	87.52%	86.90%	<0.001
FSH/LH ratio	0.627	0.605-0.648	1.54	81.70%	42.41%	<0.001

AMH, anti-müllerian hormone; AFC, antral follicle count; ROC, receiver-operator characteristic curve; AUC, the area under the curve. ^b^Binomial exact test.

For predicting POI, AMH also showed the highest accuracy (AUC 0.944, P<0.001), with the sensitivity and specificity of 92.46% and 90% respectively. AFC (AUC 0.927, P<0.001) and inhibin B (AUC 0.902, P<0.001) had slightly lower but comparable performance, but FSH/LH ratio showed the most unsatisfactory predictive value on POI (AUC 0.627, P<0.001). The cutoff value for POI diagnosis was 0.250 ng/mL for AMH, 3 for AFC, 19.08pg/mL for inhibin B and 1.54 for FSH/LH ratio, respectively (**Table 3** and **Figure 2C**).

### Ovarian Reserve Markers in POI Patients Within Different Etiologies

The causes for 601 POI patients included were assessed and consisted of genetic (n=61, 10.15%), iatrogenic (n=35, 5.82%), autoimmune (n=102, 16.97%) and idiopathic (n=403, 67.05%) (**Table 4** and **Supplemental Table 3**). No differences existed in age at diagnosis, BMI, and age at amenorrhea among four etiological subgroups (P>0.05). However, patients with genetic anomalies experienced later menarche and a trend toward earlier irregular menstruation. Interestingly, of all ovarian reserve markers, there were significant differences of AMH (P=0.011), inhibin B (P=0.003) and AFC (P<0.001) among different etiological groups. AMH level in iatrogenic POI (0.080 [0.076, 0.33] ng/mL) was significantly higher than that of genetic (0.076 [0.072, 0.083] ng/mL, P=0.022) and autoimmune POI (0.078 [0.069, 0.083] ng/mL, P=0.016) but comparable with that of idiopathic POI (0.079 [0.071, 0.090] ng/mL, P=0.108). The concentration of inhibin B was significantly lower in patients of genetic POI than that of others (P<0.05). AFC had the same distribution pattern with inhibin B except for no difference between genetic and autoimmune POI.

**Table 4:** Characteristics of POI patients within different etiologies.

Characteristics	Genetic (n=61)	Iatrogenic (n=35)	Autoimmune (n=102)	Idiopathic (n=403)	*P*
Age at diagnosis (y)	29.16 ± 4.22	30.11 ± 3.79	29.90 ± 4.30	29.71 ± 4.05	0.648^f^
BMI (kg/m^2^)	22.30 ± 2.98	21.88 ± 2.65	22.52 ± 2.98	22.80 ± 3.46	0.327^f^
Age at menarche (y)^a^ ^,^ ^c^ ^,^ ^d^ ^,^ ^e^	15.95 ± 3.34	13.40 ± 1.56	14.81 ± 2.84	14.68 ± 2.19	<0.001^f^
Age at irregularity (y)	21.18 ± 5.28	24.41 ± 5.24	21.63 ± 6.30	21.54 ± 5.83	0.056^f^
Age at amenorrhea (y)	22.14 ± 5.08	25.11 ± 5.35	23.02 ± 6.23	23.12 ± 5.80	0.186^f^
FSH (IU/L)	63.14 ± 26.66	59.82 ± 32.99	59.43 ± 23.35	62.21 ± 27.71	0.756^f^
LH (IU/L)	30.28 ± 15.91	33.53 ± 23.43	30.59 ± 15.52	31.99 ± 16.22	0.762^f^
PRL (ng/mL)	11.26 (7.94, 16.54)	11.23 (7.86, 18.30)	10.84 (7.07, 15.12)	9.90 (7.56, 14.16)	0.240^g^
E_2_ (pg/mL)	9.08 (5.00, 23.90)	12.35 (6.53, 24.78)	11.50 (5.00, 28.25)	9.00 (5.00, 23.15)	0.419^g^
T (ng/dL)	18.76 ± 14.50	17.35 ± 13.55	17.53 ± 13.11	20.36 ± 16.03	0.353^f^
AMH (ng/mL)^a^ ^,^ ^d^	0.076 (0.072, 0.083)	0.080 (0.076, 0.33)	0.078 (0.069, 0.083)	0.079 (0.071, 0.090)	0.011^g^
Inhibin B (pg/mL)^a^ ^,^ ^b^ ^,^ ^c^	10 (10, 13.69)	14 (10, 16.6)	14 (10, 16.20)	13.5 (10, 16)	0.003^g^
AFC^a^ ^,^ ^c^	0 (0, 0)	1 (0, 2)	0 (0, 1)	0 (0, 1)	<0.001^g^

Data are expressed as the mean ± standard deviation or median (interquartile range).

BMI, body mass index; FSH, follicle-stimulating hormone; LH, luteinizing hormone; E_2_, estradiol; PRL, prolactin; T, testosterone; AMH, anti-müllerian.

Hormone; AFC, antral follicle count.

a P < 0.05 for the comparison between genetic POI and iatrogenic POI.

b P < 0.05 for the comparison between genetic POI and autoimmune POI.

c P < 0.05 for the comparison between genetic POI and idiopathic POI.

d P < 0.05 for the comparison between iatrogenic POI and autoimmune POI.

e P < 0.05 for the comparison between iatrogenic POI and idiopathic POI.

f One-way analysis of variance.

g Kruskal-Wallis ANOVA test.

## Discussion

POI imposes a great challenge on women’s fertility and lifelong health. However, it is highly heterogeneous both in phenotype and in etiology. Currently, it remains controversial on its nomenclature, recruitment criteria, clinical staging and early indicators for prediction. Here we have comprehensively characterized different ovarian markers at different stages of ovarian insufficiency and within different etiologies in a large cohort of POI patients. Our results indicated that ovarian function decline was a continuum and progressive progress, in analogy to a shortened chronological aging-associated menopausal transition. When ovarian dysfunction started, the ovarian reserve indicators have begun deteriorating, especially the highly sensitive markers, such as AMH, AFC, inhibin B and FSH/LH ratio; once entered the POI stage, these indicators remained stable at low levels or even undetectable. AMH *per se* showed high predictive values for both pre-POI and POI, and a combination of AMH and AFC was highly promising to predict ovarian dysfunction in advance. More interestingly, POI patients with different etiologies showed distinct characteristics of endocrine hormones, and genetic POI showed much smaller AFC and lower level of inhibin B.

Ovarian reserve mainly encompasses the quantity and quality of oocytes. It determines a woman’s reproductive potential and, subsequently, her reproductive lifespan and age of menopause onset (17). Normal ovarian function demands integrative functioning and interactive feedback of the hypothalamic-pituitary-ovarian (HPO) axis (18). Due to the decreased quantity or quality of follicles, the insufficient secretion of ovarian hormones contributed to a preferential rise in FSH over LH through negative feedback. In pre-POI stage, FSH increased much earlier and more sharply than LH, and the FSH/LH ratio thus significantly increased. Previous evidence have shown that the FSH/LH ratio was an independent factor to predict poor ovarian response and associated with poor outcomes *in vitro* fertilization (IVF) treatment (19, 20). Here our results also demonstrated its importance in predicting pre-POI and early ovarian decline.

Within the stage of early rise of FSH, AFC, inhibin B and AMH showed similar decrease pattern before the decline of E_2_, enabling them as sensitive markers in early clinical staging of ovarian decline. AFC evaluates immediate quantity of antral follicles with good inter-cycle reliability and has been reported to be positively correlated with the number of primordial follicles (6, 21, 22). While representing the gonadotrophin-responsive antral follicle pool, inhibin B selectively inhibits pituitary FSH over LH, potentiates FSH withdrawal from non-dominant follicles and facilitates the development of a single dominant ovulatory follicle (23, 24). Both indicators showed ~ 2 folds decrease from NOR to pre-POI and provided acceptable accuracy for pre-POI prediction. The early FSH rise was probably attributed to decreased negative feedback of inhibin B decline in early follicular phase from a smaller pool of the pre-antral and early antral follicles remaining in the ovaries. Interestingly in our cohort of pre-POI, AMH declined by 5-6 folds compared to NOR and showed the highest single predictive value for pre-POI, suggesting a high sensitivity and specificity in the assessment of early ovarian dysfunction. Produced by preantral and small antral follicles, AMH can reflect more completely the size of primordial follicle pool and the number of remaining follicles. It can restrain the initial resting follicle recruitment and decreases the FSH-responsiveness of growing follicles, thus retarding the rate of follicles depletion (25, 26). Serum AMH, along with AFC, has a high sensitivity and specificity to detect the quantitative aspects of ovarian reserve, and is the most reliable contemporary ovarian reserve tests (ORT) employed today in clinical practice (27). Consistent with our data, Knauff et al. (11) found that compared with inhibin B and AFC, AMH was more consistently correlated with the clinical degree of follicle pool depletion in young women presenting with elevated FSH levels.

Currently, except for basal FSH, no standardized reference or cutoff value is available for pre-POI diagnosis (3). In our study, the ROC curve analysis revealed an area under the curve of 0.932, which implies a good discriminatory performance, and suggests that a threshold AMH value of 1.211 ng/mL would probably be a reasonable compromise for discriminating pre-POI from NOR women. Whereas both low AFC of 5 follicles and low inhibin B of 31.74 pg/mL had high specificity for predicting pre-POI, but their clinical significance was limited by its low sensitivity. Consistently, the 2016 POSEIDON criteria adopted the thresholds of AMH=1.2 ng/mL and AFC=5 for the grouping of poor ovarian response (POR) (28, 29). Although distinct concepts and diagnosis indicated, pre-POI and POR could contribute to each other due to diminished ovarian reserve, implying the clinical significance and applicability of the reference thresholds. For multiple prediction models, AMH along with AFC showed a better predictive value on pre-POI. While inhibin B and FSH/LH ratio had no additive or synergistic effects, which therefore further highlighted the importance of AMH and AFC in the very early stage of ovarian insufficiency. Although there is insufficient evidence to recommend any ovarian reserve test as a sole criterion for the use of ART (5), our quantitative changes and cutoff values in ovarian reserve markers provided a critical reference for early ovarian insufficiency, which would greatly facilitate to identify patients of high risks and timely guide family planning and fertility intervention in clinical practice. Unfortunately, the ovarian reserve markers normally change with chronological age. The age-specific cutoff values were not available and further prospective longitudinal study is warranted to confirm the predictive role of different indicators.

It was generally considered that E_2_ played a critical role in the negative feedback for FSH secretion. However, at the initial phase of ovarian insufficiency, the monotropic rise in FSH cannot be merely explained by E_2_ decrease. Estradiol levels remained unchanged or slightly elevated in early ovarian dysfunction (30–32). Consistently, no difference of E_2_ levels between women with pre-POI and NOR was revealed in this study. The compensatory HPO-axis and intra-ovarian mechanisms are operative early in ovarian aging. Lower levels of AMH in conjunction with elevated levels of FSH drive increased recruitment of the resting follicles into the growing pool. Although it contributes to accelerated follicle depletion, the increased growing follicles and continued follicle development could also maintain both estradiol levels and reproductive cycles, and serve to extend fertility and reproductive competence (33, 34). Therefore, basal estradiol level may fluctuate for variable periods of time, and it alone should not be used to predict pre-POI.

With ongoing follicle loss, the above-mentioned compensatory hormonal mechanisms are no longer adequate; follicle development becomes unpredictable, serum inhibin B and estradiol levels continue to decrease, resulting in a dramatic increase of FSH, an accelerated follicle depletion occurs (34). At this stage, oligomenorrhea or amenorrhea occurred, signifying the onset of POF. As expected, all patients with early POI already exhibited typical endocrine profiles with continuously elevated FSH and decreased E_2_. The concentrations of AMH and inhibin B were critically low or undetectable as reported previously (35), and the presence of growing follicles was found in only 34.28% of POI patients. Importantly compared with early POI, patients with POF only showed further increased LH and decreased E_2,_ whereas no difference in any sensitive ovarian markers, including AMH, Inhibin B, AFC, and FSH/LH ratio. Therefore, the sensitive ovarian reserve markers have achieved a plateau once POF occurred and their predictive advantage is reflected at the very early stage of ovarian decline.

Consistent with our previous study, women with POF experienced delayed menarche and thereafter established normal periods (13, 36). We also found that women experienced amenorrhea within two years, more than 65% within one year, after irregularity occurred, highlighting the rapid decline of ovarian function during POI progress (13). Of note, 31.95% of patients with pre-POI already exhibited irregular menstruation. Whether they are more likely to develop into POI needs long term follow-up, and corresponding intervention and fertility guidance are warranted. Another concern was the delayed diagnosis. It took approximately 6-7 years for a confirmed diagnosis of POI after amenorrhea. Although it has been reported that 5-10% of cases experienced intermittent and unpredictable resumption of ovarian activity, ovulation or spontaneous pregnancy, even years after diagnosis, occasionally occurs (37). The resumption activity is extremely subtle and hard to catch. Up to now, there are no effective treatments to restore ovarian function or improve fertility (38). A delay in diagnosis of POI, as evidenced by elevated FSH or amenorrhea, might place young women at increased risk of developing POF. Therefore, early evaluation and intervention on ovarian dysfunction according to early biochemical changes is of great significance. A standardized staging system with correct terminology for clinical assessment needs to be established based on longitudinal studies of women across the ovarian insufficiency spectrum.

POI is highly heterogeneous in etiologies, and the correlation of phenotypes and different causes currently remains poorly-uncovered. In this study, patients with genetic anomalies had the most severe defect in ovarian function, distinct from that of autoimmune or iatrogenic induced. In addition to menses abnormality, the genetic POI patients also had significantly lower inhibin B and AFC than those of other etiologies. A longitudinal study is needed to confirm the predictive value of inhibin B for the diagnosis of genetic POI. The pathogenesis and progression of autoimmune POI was assumed distinct from those of other etiologies, with follicular theca cells selectively destructed while the function of granulosa cells preserved (39). Theca cell impairment resulted in decreased estradiol synthesis and subsequent increased FSH, which stimulated the viable granulosa cells to produce more inhibin B, and the preserved ovarian follicle pool contributed to the normal AMH range. Falorni *et al.* have found that both inhibin B and AMH were significantly higher in autoimmune POI than idiopathic POI (14, 15). On the contrary, Luborsky *et al.* found no difference in inhibin B between women with and without positive ovarian antibodies (40). Similarly, the level of inhibin B and AMH was comparable between autoimmune POI and idiopathic POI patients in our study. The different recruitment criteria for autoimmune POI might explain the discrepancy. The presence of steroid-cell autoantibodies (StCA) directed against steroidogenic cells or enzymes were defined as autoimmune POI in Falorni’s studies (14, 15). While in the current study, patients with concomitant autoimmune disease, such as psoriasis, rheumatoid arthritis or thyroid autoimmunity (positive TPOAb or TGAb) were included as autoimmune POI. Given the lack of reliable and effective monitoring or diagnostic indicators for autoimmune POI currently (41), future researches to characterize the specific ovarian markers for autoimmune POI with more definite diagnostic criteria are needed.

Of note, although providing hints, our cross-sectional analysis in the current study could not exactly elucidate the progression of ovarian insufficiency. Further prospective longitudinal studies are warranted to confirm the predictive role of different indicators. Given that FSH is the single ovarian reserve marker currently used for POI defining and disease subgrouping in our study, our results, although not perfectly, could provide some information on their performance and corresponding cutoff values of other indicators inhibin B, AFC, FSH/LH and AMH, which will facilitate the identification of patients with high risks and benefit the timely fertility guidance during clinical practice.

## Conclusion

Our study depicted the dynamic changes of ovarian reserve markers in POI patients with different progressive stages and various etiologies, which provides essential evidence to confirm the high heterogeneity of POI in phenotype and etiology. The quantitative changes and cutoff values of AMH and AFC in predicting pre-POI provide new insights into the standardized staging, prediction and early diagnosis of POI. Future prospective, longitudinal cohort studies are warranted to confirm predictors and to develop strategies for fertility improvement in POI.

## Data Availability Statement

The raw data supporting the conclusions of this article will be made available by the authors, without undue reservation.

## Ethics Statement

The studies involving human participants were reviewed and approved by the Ethics Committee of Reproductive Medicine of Shandong University. The patients/participants provided their written informed consent to participate in this study.

## Author Contributions

XJ, TM, YZ, LZ, WL, and PL recruited subjects, collected data, and conducted data analysis. XJ and TM drafted the manuscript. YQ revised the manuscript critically for intellectual content. All authors contributed to the article and approved the submitted version.

## Funding

This work was supported by the National Key Research and Developmental Program of China (2018YFC1003803, 2017YFC1001100), the National Natural Science Foundation of China (81971352, 82071609), the Science Foundation for Distinguished Young Scholars of Shandong (JQ201720), and the Young Scholars Program of Shandong University.

## Conflict of Interest

The authors declare that the research was conducted in the absence of any commercial or financial relationships that could be construed as a potential conflict of interest.
