# Supplementary material for: Ovarian Reserve Markers in Premature Ovarian Insufficiency: Within Different Clinical Stages and Different Etiologies
Source: Front Endocrinol (Lausanne). 2021 Mar 18;12:601752. doi: 10.3389/fendo.2021.601752 (PMC8015703; doi:10.3389/fendo.2021.601752)
Supplement: Supplementary file 1 [file DataSheet_1.docx]

**Supplementary Table 1. The pairwise comparison among women within different stages of ovarian insufficiency.**

|  |  |  | **Age at**  **diagnosis** | **BMI** | **Age at**  **menarche** | **Age at**  **irregularity** | **FSH** | **LH** | **FSH/LH** | **E2** | **T** | **AMH** | **Inhibin B** | **AFC** |
| --- | --- | --- | --- | --- | --- | --- | --- | --- | --- | --- | --- | --- | --- | --- |
| **NOR** | vs | **pre-POI** | - | P>0.999 | P>0.999 | - | P<0.001 | P<0.001 | P<0.001 | P>0.999 | P=0.780 | P<0.001 | P<0.001 | P<0.001 |
|  |  | **early POI** | - | P=0.626 | P>0.999 | - | P<0.001 | P<0.001 | P<0.001 | P<0.001 | P=0.040 | P<0.001 | P<0.001 | P<0.001 |
|  |  | **POF** | - | P=0.318 | P<0.001 | - | P<0.001 | P<0.001 | P<0.001 | P<0.001 | P<0.001 | P<0.001 | P<0.001 | P<0.001 |
| **pre-POI** | vs | **early POI** | P<0.001 | P=0.939 | P>0.999 | P=0.100 | P<0.001 | P<0.001 | P=0.531 | P<0.001 | P=0.236 | P<0.001 | P<0.001 | P<0.001 |
|  |  | **POF** | P<0.001 | P=0.474 | P<0.001 | P=0.026 | P<0.001 | P<0.001 | P=0.005 | P<0.001 | P=0.015 | P<0.001 | P<0.001 | P<0.001 |
| **early POI** | vs | **POF** | P=0.424 | P=0.044 | P=0.012 | P>0.999 | P<0.001 | P<0.001 | P=0.026 | P=0.002 | P=0.996 | P>0.999 | P>0.999 | P>0.999 |

Abbreviations: BMI, body mass index; FSH, follicle-stimulating hormone; LH, luteinizing hormone; FSH/LH, FSH/LH ratio; E2, estradiol; PRL, prolactin; T, testosterone; AMH, anti-Müllerian hormone; AFC, antral follicle count

**Supplementary Table 2. The pairwise comparison of different predictive models of pre-POI prediction.**

|  |  |  | P value |
| --- | --- | --- | --- |
| AMH | vs | AFC | P<0.0001 |
|  |  | Inhibin B | P<0.0001 |
|  |  | FSH/LH | P<0.0001 |
|  |  | AMH + AFC | P<0.0001 |
|  |  | AMH + FSH/LH | P=0.3149 |
|  |  | AMH + Inhibin B | P=0.7148 |
| AFC | vs | Inhibin B | P<0.0001 |
|  |  | FSH/LH | P<0.0001 |
|  |  | AMH + AFC | P<0.0001 |
|  |  | AFC + FSH/LH | P<0.0001 |
|  |  | AFC + Inhibin B | P<0.0001 |
| FSH/LH | vs | Inhibin B | P=0.0288 |
|  |  | AMH + FSH/LH | P<0.0001 |
|  |  | AFC + FSH/LH | P<0.0001 |
|  |  | FSH/LH + Inhibin B | P=0.0935 |
| Inhibin B | vs | AMH + Inhibin B | P<0.0001 |
|  |  | AFC + Inhibin B | P<0.0001 |
|  |  | FSH/LH + Inhibin B | P<0.0001 |
| AMH + AFC | vs | AMH + AFC + Inhibin B | P=0.6846 |
|  |  | AMH + AFC + FSH/LH | P=0.9166 |

Abbreviations: AMH, anti-Müllerian hormone; AFC, antral follicle count

**Supplementary Table 3. The pairwise comparison among women with different etiologies of POI.**

|  |  |  | **Age at menarche** | **AMH** | **Inhibin B** | **AFC** |
| --- | --- | --- | --- | --- | --- | --- |
| **Genetic** | vs | **Iatrogenic** | P<0.001 | P=0.022 | P=0.036 | P<0.001 |
|  |  | **Autoimmune** | P=0.159 | P>0.999 | P=0.002 | P=0.073 |
|  |  | **Idiopathic** | P=0.031 | P=0.905 | P=0.007 | P=0.002 |
| **Iatrogenic** | vs | **Autoimmune** | P=0.002 | P=0.016 | P>0.999 | P=0.157 |
|  |  | **Idiopathic** | P<0.001 | P=0.108 | P>0.999 | P=0.269 |
| **Autoimmune** | vs | **Idiopathic** | P=0.998 | P=0.794 | P>0.999 | P>0.999 |

Abbreviations: AMH, anti-Müllerian hormone; AFC, antral follicle count
